# Visceral obesity anthropometric indicators as predictors of acute pancreatitis severity

**DOI:** 10.3389/fmed.2025.1536090

**Published:** 2025-07-11

**Authors:** Kaier Gu, Wenxuan Shang, Dingzhou Wang

**Affiliations:** ^1^Department of Internal Medicine, Shaoxing Maternity and Child Health Care Hospital, Shaoxing, China; ^2^Department of Cardiology, The First Affiliated Hospital of Wenzhou Medical University, Wenzhou, China; ^3^Department of Ultrasonography, The First Affiliated Hospital of Wenzhou Medical University, Wenzhou, China

**Keywords:** acute pancreatitis, anthropometric indicator, visceral adiposity index, organ failure, severity, machine learning algorithm

## Abstract

**Background:**

Acute pancreatitis (AP) severity assessment upon admission is crucial for prognosis, yet existing clinical scoring systems have limitations like delayed results, complexity, or low sensitivity. Obesity correlates with AP severity, but traditional body mass index (BMI) fails to accurately reflect visceral fat distribution. Although anthropometric indicators for visceral obesity offer alternatives, their predictive value for AP severity across all etiologies is poorly studied.

**Methods:**

This retrospective cohort study analyzed 629 AP patients admitted to a tertiary hospital (2016–2023). Patients were classified as mild AP (MAP, *n* = 531) or moderately severe/severe AP (MSAP/SAP, *n* = 98) based on organ failure (modified Marshall score ≥ 2). Eleven anthropometric indicators and six clinical scoring systems were evaluated. Patients were randomly divided into training group (*n* = 441) and validation group (*n* = 188). LASSO regression identified key predictors from 37 clinical variables. Six machine learning (ML) models were built and evaluated using receiver operating characteristic (ROC) analysis, area under the ROC curve (AUC), calibration curves, and decision curve analysis (DCA).

**Results:**

Nine anthropometric indicators [waist circumference, body roundness index, BMI, conicity index, lipid accumulation products (LAP), waist triglyceride index (WTI), cardiometabolic index (CMI), visceral adiposity index (VAI), chinese visceral adiposity index] and all clinical scoring systems (Ranson score, Glasgow score, SIRS, BISAP, APACHE II, JSS) significantly differed between MAP and MSAP/SAP groups (*p* < 0.05). VAI demonstrated the highest predictive AUC among anthropometric indicators (0.737 vs. SIRS 0.750, JSS 0.815), but superior to Ranson score, Glasgow score, BISAP, and APACHE II. LAP, WTI, and CMI also showed strong AUCs (0.729, 0.722, 0.736 respectively). LASSO selected 15 variables. Among ML models, XGBoost model performed best on the validation group (AUC = 0.878), and relatively good calibration curve and DCA results.

**Conclusion:**

VAI, CMI, LAP, and WTI are independent predictors of AP severity, with VAI showing the highest individual predictive capability among them. The XGBoost model, incorporating VAI and routinely available clinical variables, achieved excellent performance (AUC = 0.878) for early severity assessment, offering a potentially rapid and cost-effective clinical tool. This supports the utility of visceral obesity anthropometric indicators and ML models for improving early risk stratification in AP.

## 1 Introduction

Acute pancreatitis (AP) is a common inflammatory disorder of the digestive system (1), characterized by the abnormal activation of pancreatic enzymes. Worldwide, the incidence of AP is 34 cases per 100,000 individuals, and in certain regions, it is more than twice that (2). Over the past few years, there has been a notable enhancement in living standards, the incidence of AP has been steadily rising, estimated to increase by approximately 3% on average per year between 1961 and 2016 (3). AP is characterized by different degrees of prognosis. Around 80% of patients afflicted with AP present with a mild form, featuring solely pancreatic edema. This often manifests as a self-limiting disorder and holds a favorable prognosis (4). In contrast, 20% of patients with AP progress to severe forms, which might give rise to pancreatic necrosis, peritonitis, and systemic multi-organ dysfunction, accompanied by a mortality rate as elevated as 20–40% (5, 6). Therefore, evaluating the severity of AP in the initial phases of hospitalization is of considerable significance. Early intervention for high-risk patients is a necessary condition for reducing mortality and improving prognosis (7). Currently, the prediction and evaluation of the severity of AP disease are mainly conducted through clinical scoring systems, including the Ranson score, Glasgow score, systemic inflammatory response syndrome (SIRS), bedside index for severity in acute pancreatitis (BISAP), acute physiology and chronic health evaluation II (APACHE II), and Japanese severity score (JSS). However, they have certain limitations in application (8). The Ranson score and Glasgow score require more than 48 h to obtain the results (9, 10). The variables of the SIRS score include vital signs, which change constantly, thus requiring repeated evaluations (11). The BISAP score uses only five variables to predict the severity of AP patients within 24 h after admission, and its scoring sensitivity is relatively low (12, 13). The APACHE II score is designed for critically ill patients in the ICU. Multiple laboratory indicators among the variables cannot be routinely obtained within 24 h after admission, and the score is complex to use and exhibits a relatively elevated false positive rate (14). The JSS is composed of five clinical signs, 10 blood tests, CT manifestations, SIRS, and age. With complex parameters, it is restricted to a certain extent in clinical use. Consequently, there is an urgent necessity to explore novel predictive indicators that are more readily obtainable in the early stage of hospitalization, simpler to utilize, and highly accurate for identifying high-risk AP patients. This is of utmost significance for optimizing clinical decision-making and enhancing patient prognosis. Clinical evidence links obesity to AP severity (15). Body mass index (BMI) has been widely used to quantify obesity and correlate body size with fat distribution. However, BMI has limitations. In young, muscular individuals, high BMI may reflect increased muscle mass rather than obesity (16). In the elderly, declining muscle mass reduces BMI’s accuracy for obesity assessment (17). Additionally, BMI ignores variations in body fat distribution, especially visceral fat, which is closely tied to metabolic and inflammatory processes (18–20). In order to more precisely characterize varying degrees of obesity, particularly to mirror the distribution of visceral fat, several novel anthropometric indicators targeting visceral obesity have been recently introduced. These include body roundness index (BRI) (21), a body shape index (ABSI) (22), conicity index (23), weight-adjusted waist index (WWI) (24), lipid accumulation product (LAP) (25), waist triglyceride index (WTI) (26), cardiometabolic index (CMI) (27), visceral adiposity index (VAI) (28), and Chinese visceral adiposity index (CVAI) (29).

Currently, there is a paucity of studies examining the relationship between anthropometric indicators and the severity of AP. However, to date, the research regarding the correlation between these novel anthropometric indices and the severity of AP remains remarkably limited. Existing investigations have predominantly centered on specific etiologies, such as hyperlipidemic pancreatitis (HLAP) (30, 31). There is a dearth of systematic assessments encompassing the entire spectrum of AP patient populations across all etiologies. More critically, whether these novel anthropometric indices can function as straightforward, cost-effective independent predictors or complementary tools to accurately identify the severity of AP and the risk of adverse prognoses during the early stage of hospitalization has yet to be comprehensively explored and validated.

To address these gaps, this study endeavors to bridge the aforementioned knowledge gaps. Specifically, it systematically probes into the associations between a series of novel anthropometric indices and the disease severity among patients with AP across all etiologies. Simultaneously, this research assesses the latent value of these indices in predicting disease severity in AP patients during the early stage of hospitalization. The overarching objective of this study is to furnish clinical practice with a more expedient, expeditious, and potentially more precise adjunctive tool. This tool can enable the early identification of high-risk AP patients, thereby guiding more timely interventions.

## 2 Materials and methods

### 2.1 Study population

This study conducted a retrospective analysis of the AP patients who admitted to the Department of Gastroenterology at the First Affiliated Hospital of the Wenzhou Medical University from January 2016 to December 2023. The study was approved by the Ethics Committee of the First Affiliated Hospital of Wenzhou Medical University and conducted in accordance with the principles of the Declaration of Helsinki. This research constituted a retrospective, observational cohort study, wherein data were anonymized and aggregated prior to access and analysis. Consequently, the Ethics Committee granted a waiver for informed consent from all participants (Ethical Reference Number: 2025-re-031).

Inclusion criteria: meeting the AP diagnostic criteria. Exclusion criteria: (1) AP caused by ERCP, pancreatic trauma, or pancreatic surgery; (*n* = 129); (2) Ages below 18 years or above 80 years (*n* = 157); (3) Time from onset to admission more than 72 h (*n* = 802); (4) Other possible causes of pancreatic disease (chronic pancreatitis, pregnancy-related pancreatitis, or pancreatic cancer); a history of previous pancreatic-related surgery (*n* = 57); (5) Previous serious heart, lung, liver, and kidney diseases (*n* = 38); (6) A history of immune or blood disorders (*n* = 49); (7) A history of malignant tumors or previous chemotherapy or radiation therapy (*n* = 91); (8) Stay in the hospital for less than 48 h (*n* = 18); (9) Inadequate clinical records or missing medical records (*n* = 838). A total of 629 patients with AP were included in this study based on the predetermined inclusion and exclusion criteria.

**Figure 1:**
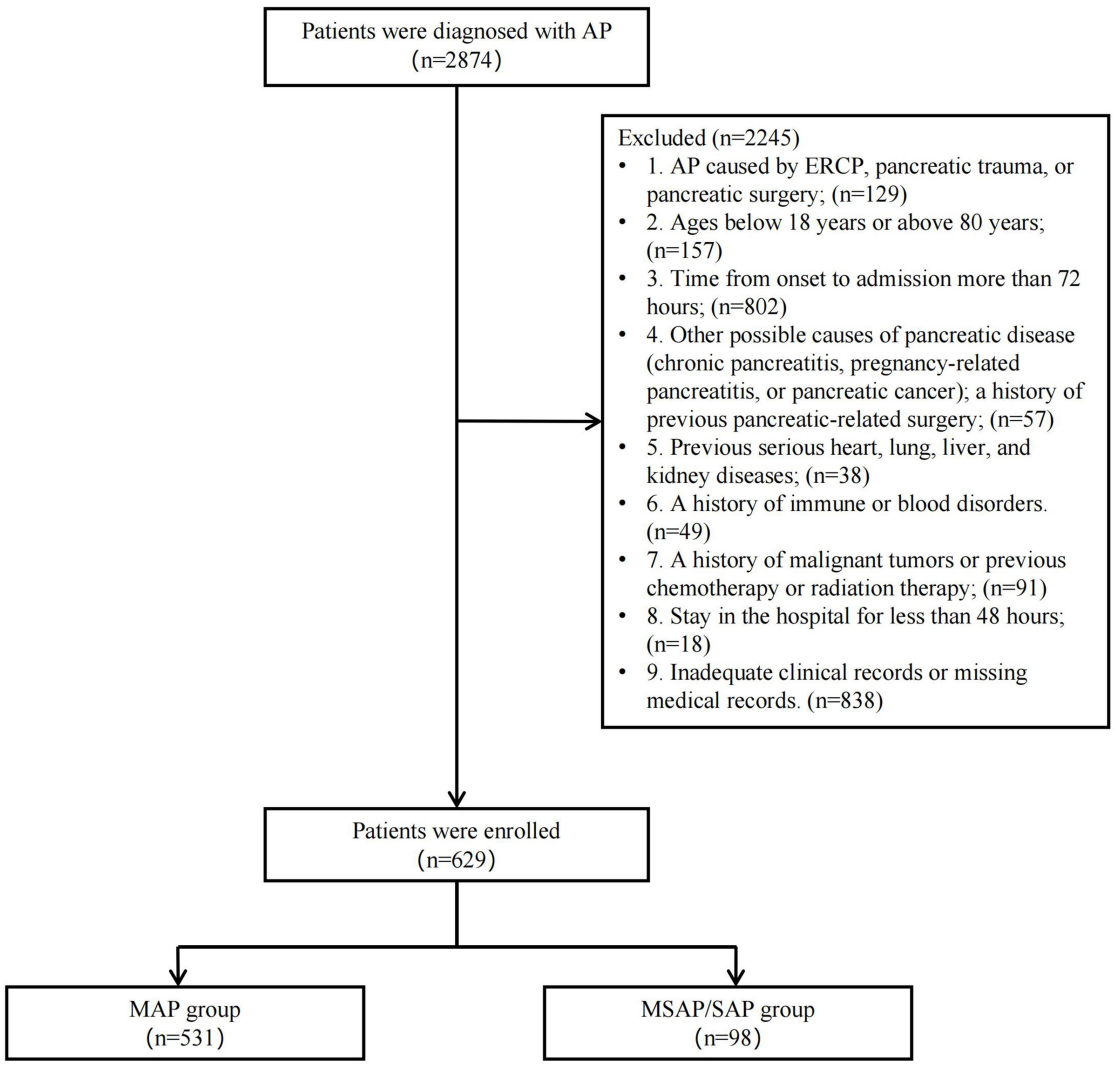
Flowchart of patient inclusion and exclusion.

Patients were divided into two groups based on the incidence of organ failure (OF): the MSAP/SAP group (*n* = 98) and the MAP group (*n* = 531). The selection process is shown in Figure 1.

### 2.2 Diagnostic criteria for AP

Two or more of the following three requirements must be met in order to diagnose AP, under the 2012 revisions to the Atlanta criteria (32): (a) Persistent upper abdominal pain; (b) Characteristic imaging findings of AP; (c) Serum amylase and/or lipase levels were elevated to at least three times the upper limit of normal.

The severity of the disease is classified as mild or moderate/severe based on whether OF and local and/or systemic complications occur. The diagnosis of OF is based on the modified Marshall scoring system, namely the assessment of the respiratory, renal, and cardiovascular systems. The definition of OF is a modified Marshall score of at least two for any one of the three systems. Local complications include acute peripancreatic fluid collections, acute necrotic fluid collections, pancreatic pseudocysts, walled-off necrosis, and infected pancreatic necrosis.

### 2.3 Data collection

Data were retrospectively collected from electronic medical records of the First Affiliated Hospital of Wenzhou Medical University. Based on literature and clinical experience, we selected 45 potential predictor variables, including baseline characteristics, medical history, etiology, laboratory results, and endpoint events. Baseline clinical characteristics: gender, age, BMI, waist circumference (WC). Medical history: hypertension, diabetes, fatty liver, hyperlipemia, drinking, smoking. Etiology: biliary, hyperlipemia, alcohol abuse, unknown. Laboratory findings: white blood cell (WBC), red blood cell (RBC), hemoglobin (Hb), platelet count (PLT), hematocrit (HCT), total bilirubin (TBil), albumin (Alb), alanine aminotransferase (ALT), aspartate transaminase (AST), alkaline phosphatase (ALP), blood glucose (BG), blood urea nitrogen (BUN), creatinine (Cr), potassium (K^+^), sodium (Na^+^), chlorine (Cl^−^), calcium (Ca^2+^), amylase (AMY), total cholesterol (TC), triglyceride (TG), high-density lipoprotein cholesterol (HDL), low-density lipoprotein cholesterol (LDL), lipase (LIP), C-reactive protein (CRP), thrombin time (TT), prothrombin time (PT), fibrinogen (Fib), activated partial thromboplastin time (APTT), international standardized ratio (INR), D-dimer. The initial samples were collected within 24 h of admission to minimize the potential interference from subsequent treatments. Endpoint event: whether or OF, admission to the ICU, length of stay in hospital. Based on the medical records, six commonly used clinical scoring systems for the severity of AP at admission were calculated: Ranson score, Glasgow score, SIRS, BISAP, APACHE II, and JSS.

### 2.4 The calculation of anthropometric makers for visceral obesity

All anthropometric indicators are calculated through the following standard equations:


$$\mathrm{BMI}=\frac{\text{Weight}\;(\mathrm{kg})}{\text{Height}\;{\left(m\right)}^{2}}$$

$$\mathrm{BRI}=364.2-365.5\times \sqrt{1-{\left[\frac{\frac{\mathrm{WC}\;(\mathrm{cm})}{2\pi }}{0.5\times \text{Height}\;(m)}\right]}^{2}}$$

$$\text{ABSI}=\frac{\mathrm{WC}\;(\mathrm{cm})}{\sqrt[{3}]{\mathrm{BMI}\;{\left(\mathrm{kg}/m^{2}\right)}^{2}}\times \sqrt[{2}]{\text{Height}\;(m)}}$$

$$\mathrm{CI}=\frac{\mathrm{WC}\;(m)}{0.109\times \sqrt{\frac{\text{Weight}\;(\mathrm{kg})}{\text{Height}\;(\mathrm{cm})}}}$$

$$\mathrm{WWI}=\frac{\mathrm{WC}\;(\mathrm{cm})}{\sqrt{\text{Weight}\;(\mathrm{kg})}}$$

LAP(females)=TG(mmol/L)×(WC(cm)−58)

LAP(males)=TG(mmol/L)×(WC(cm)−65)

WTI=WC(cm)×TG(mmol/L)

$$\mathrm{CMI}=\frac{\mathrm{TG}\;(\text{mmol}/L)}{\mathrm{HDL}\;(\text{mmol}/L)}\times \frac{\mathrm{WC}\;(\mathrm{cm})}{\text{Height}\;(\mathrm{cm})}$$

$$\begin{array}{c} \mathrm{VAI}\;(\text{males})=\frac{\mathrm{WC}\;(\mathrm{cm})}{39.68+1.88\times \mathrm{BMI}\;(\mathrm{kg}/m^{2})}\times \frac{\mathrm{TG}\;(\text{mmol}/L)}{1.03}\\ \times \frac{1.31}{\mathrm{HDL}\;(\text{mmol}/L)} \end{array}$$

$$\begin{array}{c} \mathrm{VAI}\;(\text{females})=\frac{\mathrm{WC}\;(\mathrm{cm})}{39.58+1.89\times \mathrm{BMI}\;(\mathrm{kg}/m^{2})}\times \frac{\mathrm{TG}\;(\text{mmol}/L)}{0.81}\\ \times \frac{1.52}{\mathrm{HDL}\;(\text{mmol}/L)} \end{array}$$

$$\begin{array}{c} \text{CVAI}\;(\text{males})=-267.93+0.68\times \mathrm{age}+0.03\times \mathrm{BMI}\;(\mathrm{kg}/m^{2})+4.00\\ \times \mathrm{WC}\;(\mathrm{cm})+22.00\times \log_{10}\mathrm{TG}\;(\text{mmol}/L)\\ -16.32\times \mathrm{HDL}\;(\text{mmol}/L) \end{array}$$

$$\begin{array}{c} \;\text{CVAI}\;(\text{females})=-187.32+1.71\times \mathrm{age}+4.32\times \mathrm{BMI}\;(\mathrm{kg}/m^{2})\\ +1.12\times \mathrm{WC}\;(\mathrm{cm})+39.67\times \log_{10}\mathrm{TG}\;(\text{mmol}/L)\\ -11.66\times \mathrm{HDL}\;(\text{mmol}/L) \end{array}$$

WC is estimated through abdominal CT imaging. The transverse and longitudinal axes of the umbilicus on abdominal CT are measured. Subsequently, WC is calculated by using the standard ellipse formula (33).


$$\mathrm{WC}=\text{elliptic coefficient}\times \frac{\text{short axis}(\mathrm{cm})+\text{long axis}(\mathrm{cm})}{2}$$

### 2.5 Data processing

Excel was used to sanitize the data. Variable with a missing data rate of more than 20% (D-dimer) was excluded; multiple imputation was employed to fill missing values for variables ranging from 5 to 20% (BG, Ca^2+^, LIP, TT, PT, FIB, APTT, INR), aiming to identify the optimal dataset for imputing these values. Variables with a missing data rate of less than 5% (WBC, RBC, Hb, PLT, HCT, TBIL, Alb, ALT, AST, ALP, BUN, Cr, K^+^, Na^+^, Cl^−^, AMY, CRP, and total length of hospital stay) were replaced with their mean values.

### 2.6 Model construction

The recruited patients were divided into two groups at random (7:3): the training group and the validation group. Models with different machine learning (ML) techniques were developed using the training group, and their performance was assessed using the validation group. The dataset was divided into two groups, training (441 patients total, 64 MSAP/SAP, and 377 MAP) and validation (188 patients total, 34 MSAP/SAP, and 154 MAP). From the training group, clinical variables were chosen using the Least Absolute Shrinkage and Selection Operator (LASSO) regression approach. The selected variables were incorporated into six distinct ML models, including K-Nearest Neighbor (KNN), Light Gradient Boosting Machine (LGBM), Logistic Regression (LR), Random Forest (RF), Support Vector Machine (SVM), and eXtreme Gradient Boosting (XGB). The models’ identification capability was assessed using the receiver operating characteristic (ROC) curve, along with the calculation of the area under the ROC curve (AUC) and confusion matrices. The calibration of the models was assessed by a calibration curve. The clinical effectiveness of the models was evaluated by decision curve analysis (DCA). The complete research flow chart is presented in Figure 1.

### 2.7 Rationality of sample size

This study is a retrospective cohort analysis, where the sample size was naturally determined according to the inclusion and exclusion criteria. Despite the absence of a prospective power analysis, the adequacy of the sample size is evidenced by the following aspects: 1. Epidemiological Rationality: The proportion of patients with MSAP/SAP was 15.6% (98/629), which is in line with the reported international incidence range of SAP (10–20%) (34). 2. Power Verification: A post-hoc power analysis was performed using G*Power 3.1 software (parameters: significance level *α* = 0.05, effect size *d* = 0.5). The results demonstrated that the power to detect differences between groups for the key indicators (e.g., VAI) reached as high as 99.2%. 3. Result Stability: The width of the CI of the AUC for all anthropometric indicators was less than 0.2. This conforms to the stability criteria of the predictive model, suggesting that a confidence interval width < 0.2 implies a relatively minor impact of sampling variation.

### 2.8 Statistical analysis

Statistical analyses were executed by means of SPSS software (version 25.0). Categorical variables were represented as counts and percentages, and inter-group comparisons were carried out via the chi-square test. The normality of continuous variables was appraised through the Kolmogorov–Smirnov test. Variables adhering to a normal distribution were presented as mean ± standard deviation, conversely, non-normally distributed variables were depicted as median and interquartile range. The Spearman rank correlation coefficient was enlisted to scrutinize the relationship between two continuous variables with non-normal distributions. The predictive capabilities of anthropometric indicators and clinical scoring systems were assessed by ROC curves and the calculation of the AUC. The optimal cut-off values of each index for predicting the severity of AP were determined by maximizing Youden’s Index (J), where J = sensitivity + specificity - 1. This cut-off point corresponds to the point on the ROC curve that is closest to the upper left corner, representing the best balance between sensitivity and specificity. A *p* value less than 0.05 was regarded as statistically substantive.

## 3 Results

### 3.1 Characteristics of participants

To study the differences in baseline characteristics of patients with different outcomes of AP, a retrospective analysis was performed on 629 enrolled patients. Tables 1, 2 presents the differences in baseline characteristics of patients with different outcomes of AP. Among all the patients, the incidence of MSAP/SAP group was 15.6% (n = 98), including 437 males (69.48%) and 192 females (30.52%). The mean values of age, BMI, and WC of the patients were 48.16 years, 25.06 kg/m^2^, and 85.37 cm, respectively, and the median TG was 1.94 mmol/L. The ICU admission rate was 2.54%. Compared to the MAP group, MSAP/SAP patients had significantly younger age, higher BMI, WC, and TG levels, and higher rates of diabetes, fatty liver, alcohol use, smoking, and ICU admission. Laboratory findings (WBC, RBC, Hb, HCT, Alb, ALT, ALP, BG, Na^+^, Cl^−^, Ca^2+^, TC, TG, HDL, LDL, CRP, PT, Fib, INR, D-dimer) within 24 h of admission also differed significantly (*p* < 0.05). Meanwhile, no significant differences were observed in gender and the prevalence of hypertension between the MAP group and the MSAP/SAP group (*p* > 0.05).

**Table 1:** Comparison of baseline characteristics, medical history, etiology, and endpoint events between patients with MAP group and MSAP/SAP group.

Variables	Total (*n* = 629)	MAP (*n* = 531)	MSAP/SAP (*n* = 98)	*p* value
Baseline characteristics				
Gender, male, *N* (%)	437 (69.48)	367 (69.11)	70 (71.43)	0.648
Age, years (SD)	48.16 ± 13.64	48.77 ± 14.01	44.83 ± 10.94	0.002
BMI, kg/m^2^ (IQR)	25.06 (22.76, 27.68)	24.22 (22.05, 26.34)	25.39 (22.82, 27.73)	0.018
WC, cm (SD)	85.37 ± 9.37	84.86 ± 9.38	88.12 ± 8.90	0.002
Medical history				
Hypertension, *N* (%)	139 (22.1)	123 (23.16)	16 (16.33)	0.134
Diabetes, *N* (%)	167 (26.55)	123 (23.16)	44 (44.90)	<0.001
Fatty Liver, *N* (%)	372 (59.14)	295 (55.56)	77 (78.57)	<0.001
Hyperlipemia, *N* (%)	282 (44.83)	213 (40.11)	69 (70.41)	<0.001
Drinking, *N* (%)	253 (40.22)	198 (37.29)	55 (56.12)	<0.001
Smoking, *N* (%)	224 (35.61)	176 (33.15)	48 (48.98)	0.003
Etiology				<0.001
Biliary, *N* (%)	263 (41.81)	242 (45.57)	21 (21.43)	
Hyperlipemia, *N* (%)	41 (6.52)	35 (6.59)	6 (6.12)	
Alcohol abuse, *N* (%)	200 (31.80)	138 (25.99)	62 (63.27)	
Unknown, *N* (%)	125 (19.87)	116 (21.85)	9 (9.18)	
Endpoint event				
AICU, *N* (%)	16 (2.54)	3 (0.56)	13 (13.27)	<0.001
Hospital LOS, *N* (%)	6.86 (5.26, 9.82)	6.80 (5.56, 9.73)	16.79 (11.63, 23.70)	<0.001

AP, Acute pancreatitis; MAP, Mild acute pancreatitis; MSAP, Moderately severe acute pancreatitis; SAP, Severe acute pancreatitis; BMI, Body mass index; WC, Waist circumference; AICU, Admission to intensive care unit; LOS, Length of stay.

**Table 2:** Comparison of laboratory findings between patients with MAP group and MSAP/SAP group.

Variables	Total (*n* = 629)	MAP (*n* = 531)	MSAP/SAP (*n* = 98)	*p* value
WBC, 10^9^/L (IQR)	10.21 (7.66, 13.43)	10.03 (7.29, 12.48)	13.65 (9.91, 17.37)	<0.001
RBC, 10^12^/L (IQR)	4.57 (4.16, 4.96)	4.42 (4.04, 4.91)	4.91 (4.57, 5.60)	<0.001
Hb, g/L (IQR)	140 (128, 152)	137 (127, 149)	154 (134, 172)	<0.001
PLT, 10^9^/L (IQR)	204 (170, 242)	193 (157, 231)	201 (172, 253)	0.107
HCT (IQR)	0.41 (0.38, 0.45)	0.40 (0.37, 0.44)	0.44 (0.40, 0.50)	<0.001
TBil, μmol/L (IQR)	20 (15, 30)	20 (15, 41)	20 (16, 27)	0.376
Alb, g/L (SD)	37.54 ± 4.49	37.97 ± 4.30	35.26 ± 4.84	<0.001
ALT, U/L (IQR)	30 (17, 77)	40 (18, 123)	26 (14, 49)	0.014
AST, U/L (IQR)	28 (20, 58)	31 (19, 82)	38 (23, 53)	0.324
ALP, U/L (IQR)	83 (67, 109)	88 (70, 130)	63 (58, 78)	0.005
BG, mmol/L (IQR)	7.6 (5.8, 11.2)	7.6 (5.7, 11.3)	11.4 (7.2, 15.4)	<0.001
BUN, mmol/L (IQR)	4.0 (3.1, 5.3)	4.5 (3.2, 5.5)	5.7 (3.7, 7.1)	0.060
Cr, μmol/L (IQR)	65 (54, 77)	66 (54, 77)	66 (56, 89)	0.794
K^+^, mmol/L (IQR)	3.92 (3.70, 4.12)	3.90 (3.73, 4.09)	4.06 (3.70, 4.50)	0.135
Na^+^, mmol/L (SD)	136.87 ± 3.27	137.18 ± 3.00	135.21 ± 4.10	<0.001
Cl^−^, mmol/L (IQR)	103 (100, 105)	103 (101, 105)	102 (99, 106)	0.004
Ca^2+^, mmol/L (IQR)	2.16 (2.06, 2.24)	2.15 (2.09, 2.24)	1.86 (1.74, 2.13)	<0.001
AMY, U/L (IQR)	151 (72, 387)	225 (79, 489)	307 (143, 770)	0.093
TC, mmol/L (IQR)	5.32 (4.26, 7.14)	4.99 (4.26, 6.06)	5.71 (3.54, 9.39)	<0.001
TG, mmol/L (IQR)	1.94 (0.95, 5.79)	1.59 (0.87, 3.48)	5.27 (1.19, 11.22)	<0.001
HDL, mmol/L (SD)	1.10 ± 0.35	1.12 ± 0.33	1.02 ± 0.42	0.024
LDL, mmol/L (IQR)	3.04 (2.34, 3.90)	2.89 (2.20, 3.56)	2.93 (1.78, 4.17)	0.240
LIP, IU/L (IQR)	192 (79, 514)	215 (88, 663)	349 (89, 1,073)	0.122
CRP, mg/L (IQR)	106.6 (34.4, 192.7)	105.9 (33.7, 187.8)	205.5 (121.9, 298.1)	<0.001
TT, seconds (IQR)	16.3 (15.5, 17.1)	16.4 (15.5, 17.3)	16.7 (15.9, 17.5)	0.523
PT, seconds (IQR)	13.8 (13.2, 14.5)	13.9 (13.3, 14.6)	14.3 (13.9, 15.5)	0.002
Fib, g/L (IQR)	4.83 (3.77, 6.38)	4.80 (3.77, 6.41)	5.86 (4.08, 6.71)	<0.001
APTT, seconds (IQR)	37.1 (34.2, 40.3)	37.2 (34.7, 40.4)	38.1 (34.1, 41.9)	0.951
INR (IQR)	1.06 (1.01, 1.12)	1.07 (1.02, 1.15)	1.11 (1.07, 1.24)	0.003

AP, Acute pancreatitis; MAP, Mild acute pancreatitis; MSAP, Moderately severe acute pancreatitis; SAP, Severe acute pancreatitis; WBC, White blood cell; RBC, Red blood cell; Hb, Hemoglobin; PLT, Platelet counts; HCT, Hematocrit value; TBil, Total bilirubin; Alb, Albumin; ALT, Alanine aminotransferase; AST, Aspartate transaminase; ALP, Alkaline phosphatase; BG, Blood glucose; BUN, Blood urea nitrogen; Cr, Creatinine; K^+^, Potassium; Na^+^, Sodium; Cl^−^, Chlorine; Ca^2+^, Calcium; AMY, Amylase; TC, Total cholesterol; TG, Triglyceride; HDL, High-density lipoprotein cholesterol; LDL: Low-density lipoprotein cholesterol; LIP, Lipase; CRP, C-reactive protein; TT, Thrombin time; PT, Prothrombin time; Fib, Fibrinogen; APTT, Activated partial thromboplastin time; INR, International standardized ratio.

### 3.2 The correlation among anthropometric indicators

**Figure 2:**
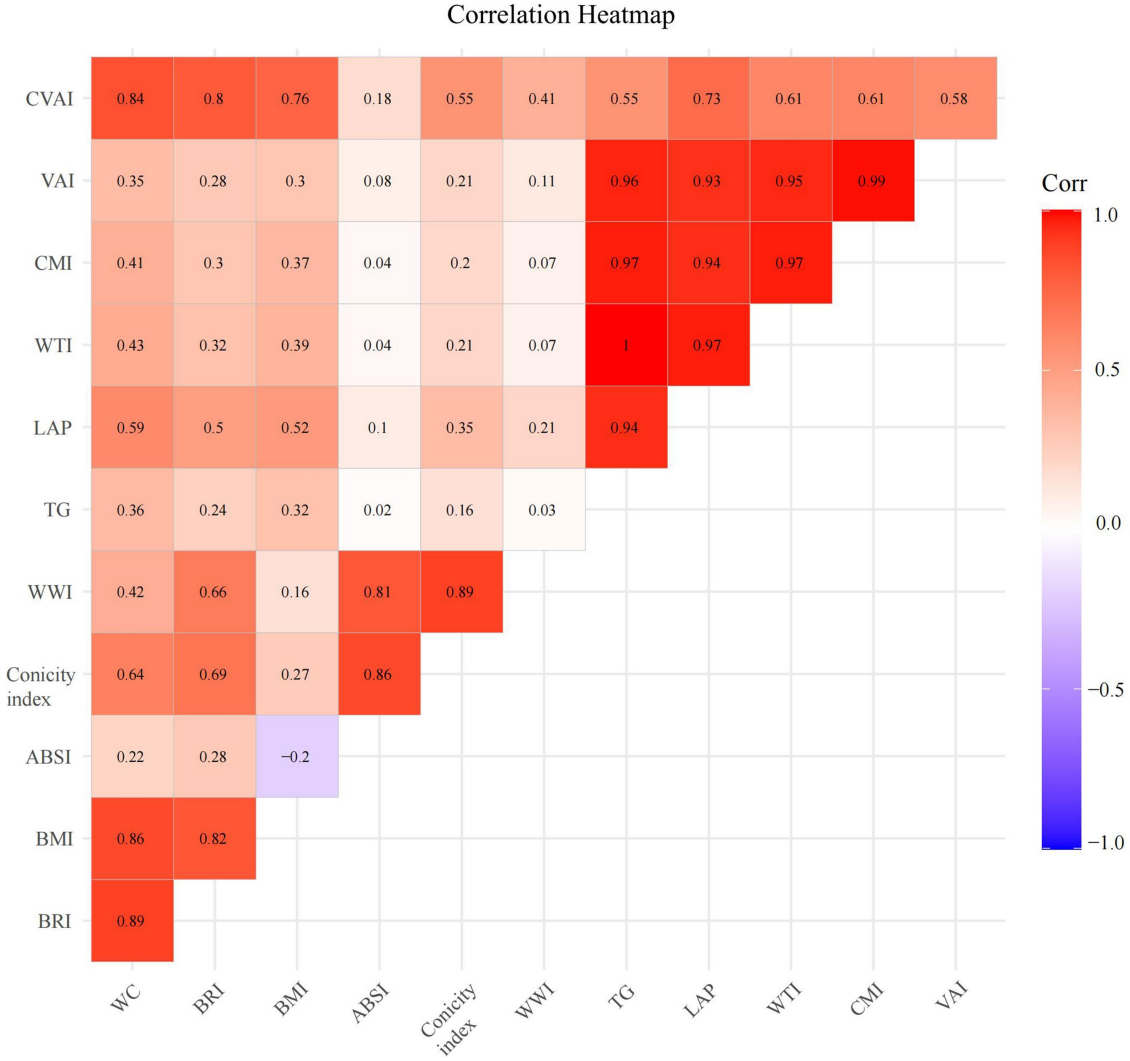
Correlation heatmap of anthropometric indicators with visceral obesity. WC, Waist circumference; BRI, Body roundness index; BMI, Body mass index; ABSI, A body shape index; TG, triglyceride; WWI, Weight-adjusted waist index; LAP, Lipid accumulation product; WTI, Waist triglyceride index; CMI, Cardiometabolic index; VAI, Visceral adiposity index; CVAI, Chinese visceral adiposity index.

**Figure 3:**
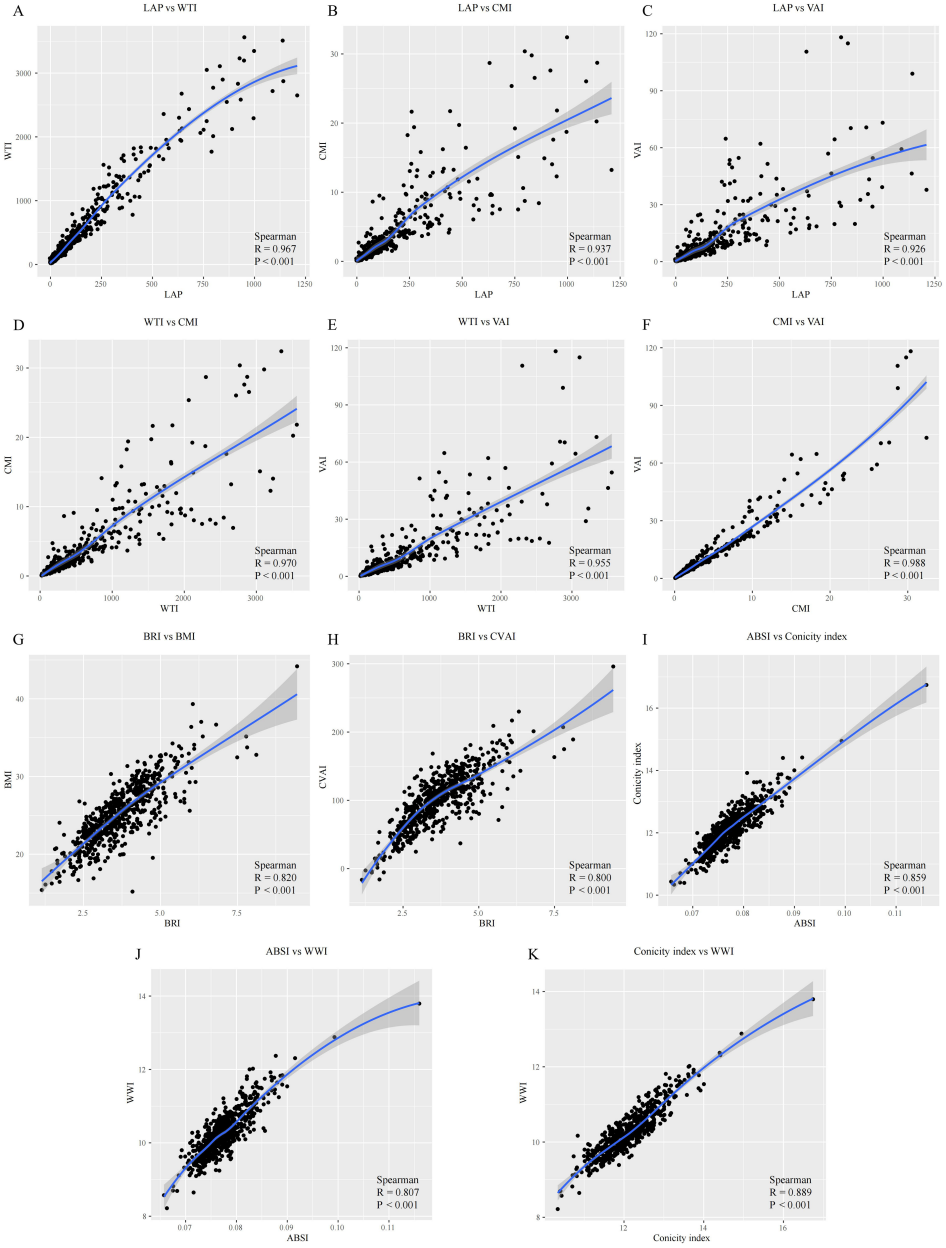
Correlation analysis of anthropometric indicators with visceral obesity. LAP, Lipid accumulation product; WTI, Waist triglyceride index; CMI, Cardiometabolic index; VAI, Visceral adiposity index; CVAI, Chinese visceral adiposity index; BRI, Body roundness index; BMI, Body mass index; ABSI, A body shape index; WWI, Weight-adjusted waist index.

To further investigate the correlation among anthropometric indicators, TG, and WC, Figure 2 presents the magnitudes of their rank correlation coefficients using a heatmap. The results demonstrated a significant positive correlation between TG and VAI, CMI, LAP, and WTI. Meanwhile, a significant positive correlation was observed between WC and BRI, BMI, and CVAI. Statistically significant correlations could be observed between any two variables among VAI, CMI, LAP, and WTI. For further analysis of the correlation among these variables, refer to Figure 3. Additionally, statistically significant correlations could also be observed between the following pairs of variables: BRI vs. BMI, BRI vs. CVAI, ABSI vs. CI, ABSI vs. WWI, and CI vs. WWI.

### 3.3 Comparison of anthropometric indicators between the MAP and MSAP/SAP groups

To explore and compare the correlations between the severity of AP disease and anthropometric indicators as well as common clinical scoring systems, this study analyzed and compared 11 anthropometric indicators and six common clinical scoring systems of patients in the MAP and MSAP/SAP groups. As demonstrated in Table 3, the severity of AP disease was significantly correlated with nine anthropometric indicators (WC, BRI, BMI, CI, LAP, WTI, CMI, VAI, and CVAI) and all six common clinical scoring systems (Ranson score, Glasgow score, SIRS, BISAP, APACHE II, and JSS) (*p* < 0.05).

**Table 3:** Comparison of novel anthropometric indicators and clinical scoring systems for different severities of AP.

Variables	Total (*n* = 629)	MAP (*n* = 531)	MSAP/SAP (*n* = 98)	*p* value
WC (SD)	85.37 ± 9.37	84.86 ± 9.38	88.12 ± 8.90	0.002
BRI (IQR)	3.57 (2.93, 4.31)	3.42 (2.88, 4.18)	3.80 (3.18, 4.45)	0.007
BMI (SD)	25.06 (22.76, 27.68)	24.22 (22.05, 26.34)	25.39 (22.82, 27.73)	0.018
ABSI (IQR)	0.0770 (0.0746, 0.0793)	0.0775 (0.0747, 0.0792)	0.0782 (0.0750, 0.0815)	0.208
Conicity index (IQR)	12.11 (11.69, 12.5)	12.08 (11.66, 12.44)	12.24 (11.86, 12.65)	0.010
WWI (SD)	10.27 ± 0.61	10.25 ± 0.62	10.36 ± 0.53	0.120
LAP (IQR)	44.87 (17.32, 143.29)	36.23 (13.34, 80.88)	130.64 (29.92, 258.45)	<0.001
WTI (IQR)	166.3 (74.79, 524.71)	129.48 (68.33, 305.62)	490.74 (107.44, 954.04)	<0.001
CMI (IQR)	0.99 (0.40, 3.24)	0.79 (0.34, 2.22)	3.90 (0.53, 5.39)	<0.001
VAI (IQR)	2.65 (1.27, 8.42)	2.30 (1.04, 5.21)	12.27 (1.56, 14.88)	<0.001
CVAI (SD)	100.99 ± 39.56	97.94 ± 39.29	117.53 ± 37.04	<0.001
Ranson score (IQR)	1.00 (0.00, 2.00)	1.00 (0.00, 2.00)	2.00 (1.00, 3.00)	<0.001
Glasgow score (IQR)	1.00 (0.00, 2.00)	1.00 (0.00, 2.00)	3.00 (2.00, 3.00)	<0.001
SIRS (IQR)	1.00 (0.00, 2.00)	1.00 (0.00, 2.00)	2.00 (1.00, 2.00)	<0.001
BISAP (IQR)	1.00 (0.00, 1.00)	1.00 (0.00, 1.00)	1.00 (1.00, 2.00)	<0.001
APACHE II (IQR)	5.00 (3.00, 7.00)	5.00 (4.00, 7.00)	8.00 (5.00, 13.00)	<0.001
JSS (IQR)	1.00 (0.00, 3.00)	1.00 (0.00, 3.00)	4.00 (2.00, 5.00)	<0.001

AP, Acute pancreatitis; MAP, Mild acute pancreatitis; MSAP, Moderately severe acute pancreatitis; SAP, Severe acute pancreatitis; WC, Waist circumference; BRI, Body roundness index; BMI, Body mass index; ABSI, A body shape index; WWI, Weight-adjusted waist index; LAP, Lipid accumulation products; WTI, Waist triglyceride index; CMI, Cardiometabolic index; VAI, Visceral adiposity index; CVAI, Chinese visceral adiposity index; SIRS, Systemic inflammatory response syndrome; BISAP, Bedside index of severity in acute pancreatitis; APACHE II, Acute physiology and chronic health evaluation II; JSS, Japanese severity score.

The nine anthropometric indicators and all six common clinical scoring systems in the MSAP/SAP group were significantly elevated compared to those in the MAP group, while the other two anthropometric indicators (ABSI and WWI) showed no significant statistical difference. This outcome indicates that anthropometric indicators may have the potential to predict the severity of AP.

### 3.4 Evaluation of the capacity of anthropometric indicators in predicting the severity of AP

**Figure 4:**
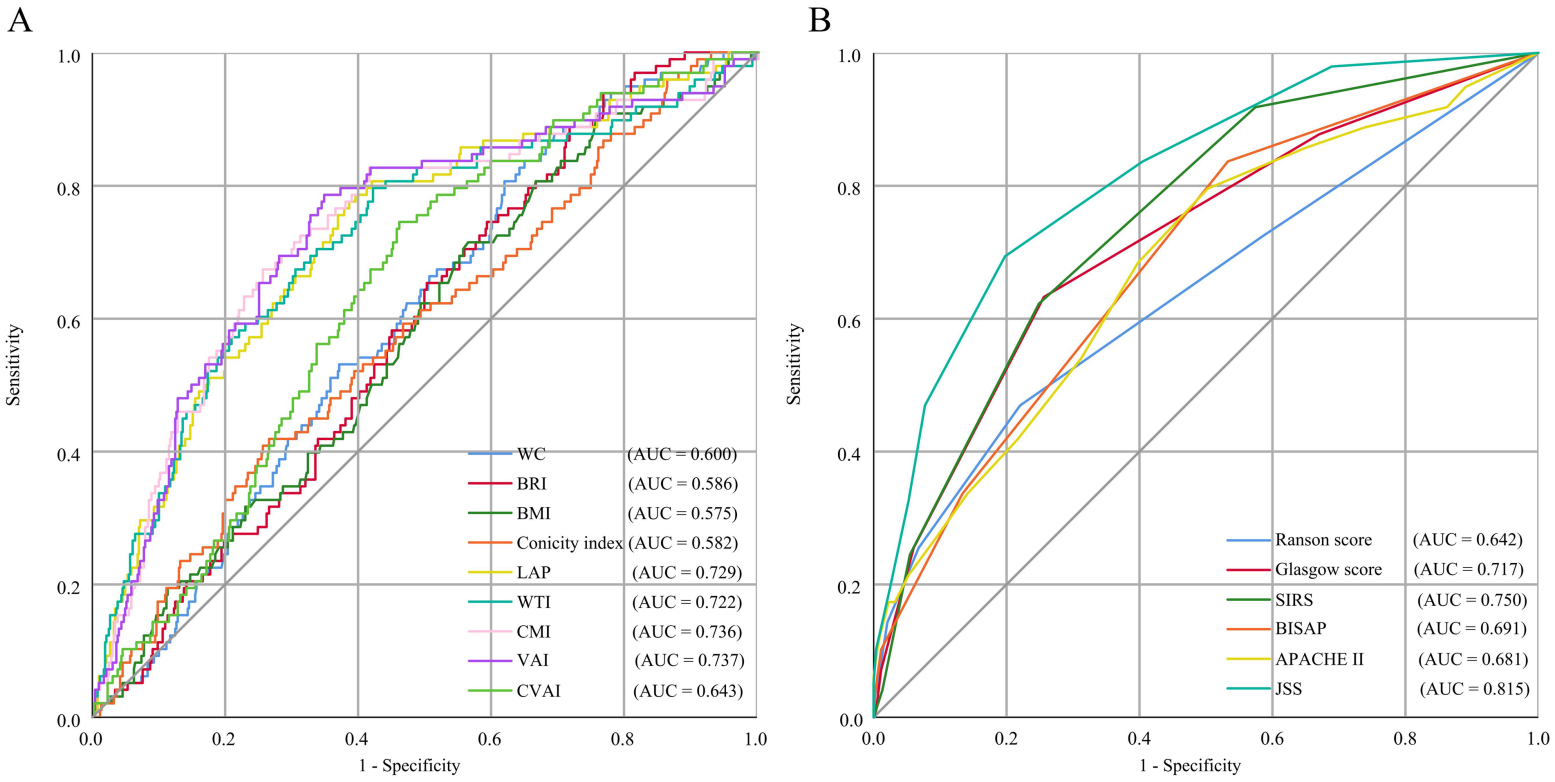
ROC curve analysis of anthropometric indicators **(A)** and clinical scoring systems **(B)**. ROC, receiver operating characteristic; AUC, area under the curve; WC, Waist circumference; BRI, Body roundness index; BMI, Body mass index; CI, Conicity index; LAP, Lipid accumulation product; WTI, Waist triglyceride index; CMI, Cardiometabolic index; VAI, Visceral adiposity index; CVAI, Chinese visceral adiposity index; SIRS, Systemic inflammatory response syndrome; BISAP, Bedside index of severity in acute pancreatitis; APACHE II, Acute physiology and chronic health evaluation II; JSS, Japanese severity score.

To compare the effectiveness of anthropometric indicators in predicting the severity of AP, this research calculated the AUC values (Table 4) and plotted the ROC curves (Figure 4) for the aforementioned nine anthropometric indicators and six commonly used clinical scoring systems. The AUC of VAI presents the best predictive performance, followed closely by CMI, LAP, and WTI. The prediction probabilities for these four anthropometric indicators were marginally lower than that of JSS, but were proximate to the SIRS score and higher than those of several other scores. This outcome further demonstrates that anthropometric indicators can be employed to prognosticate the severity of AP.

**Table 4:** Comparison of diagnostic performance for predicting different severities of AP between novel anthropometric indicators and clinical scoring systems.

Variables	AUC (95% CI)	*p* value	Cutoff value	Sensitivity	Specificity
WC	0.600 (0.545–0.655)	0.002	80.815	0.837	0.352
BRI	0.586 (0.530–0.641)	0.007	2.812	0.939	0.234
BMI	0.575 (0.516–0.634)	0.018	22.479	0.908	0.243
Conicity index	0.582 (0.521–0.643)	0.010	12.410	0.418	0.736
LAP	0.729 (0.673–0.784)	<0.001	56.222	0.786	0.606
WTI	0.722 (0.664–0.781)	<0.001	187.471	0.796	0.580
CMI	0.736 (0.679–0.793)	<0.001	2.298	0.673	0.746
VAI	0.737 (0.680–0.794)	<0.001	3.874	0.786	0.653
CVAI	0.643 (0.589–0.697)	<0.001	101.894	0.745	0.540
Ranson score	0.642 (0.576–0.707)	<0.001	1.500	0.469	0.780
Glasgow score	0.717 (0.660–0.775)	<0.001	1.500	0.633	0.744
SIRS	0.750 (0.699–0.800)	<0.001	1.500	0.622	0.751
BISAP	0.691 (0.635–0.747)	<0.001	0.500	0.837	0.467
APACHE II	0.681 (0.622–0.740)	<0.001	4.500	0.796	0.497
JSS	0.815 (0.770–0.860)	<0.001	2.500	0.694	0.802

AP, Acute pancreatitis; WC, Waist circumference; BRI, Body roundness index; BMI, Body mass index; ABSI, A body shape index; WWI, Weight-adjusted waist index; LAP, Lipid accumulation products; WTI, Waist triglyceride index; CMI, Cardiometabolic index; VAI, Visceral adiposity index; CVAI, Chinese visceral adiposity index; SIRS, Systemic inflammatory response syndrome; BISAP, Bedside index of severity in acute pancreatitis; APACHE II, Acute physiology and chronic health evaluation II; JSS, Japanese severity score.

### 3.5 Identification of clinical variables for selection

**Figure 5:**
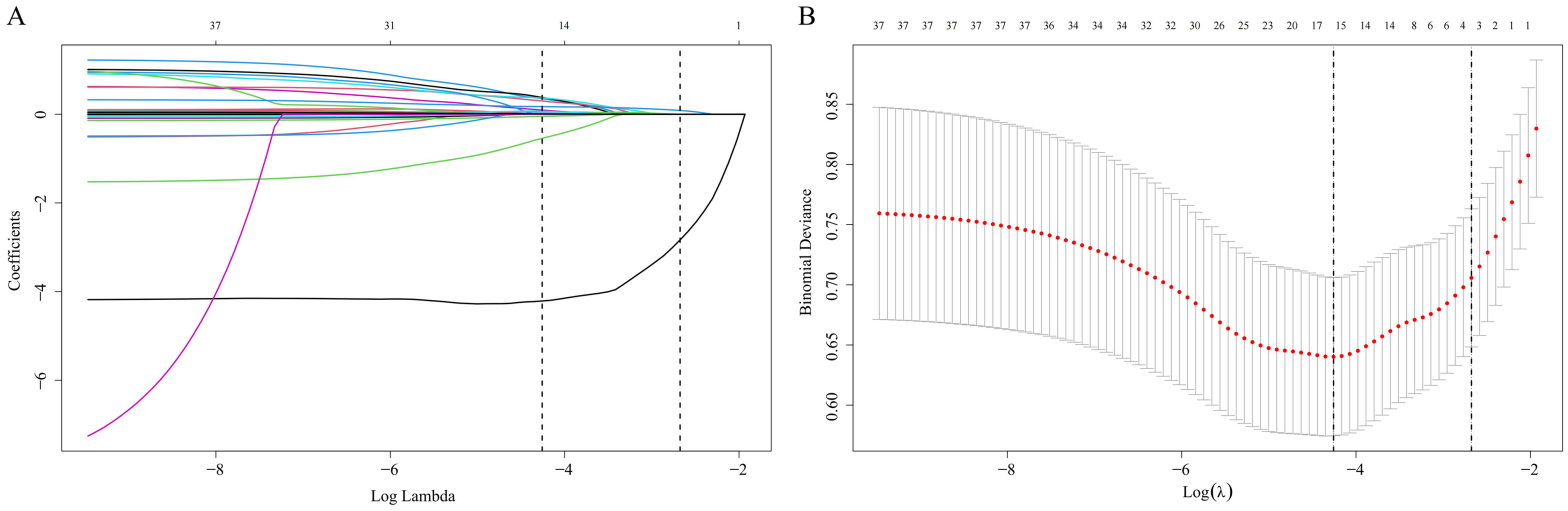
Selection of clinical variables via the LASSO regression method. **(A)** The coefficient values for 37 variables are shown in relation to log(*λ*); **(B)** The left vertical dashed line demarcates the position where the minimum cross-validation error emerges, while the right vertical dashed line specifies the minimum error plus one standard deviation. LASSO, Least Absolute Shrinkage and Selection Operator.

We constructed and verified prediction models with clinical variables encompassing anthropometric indicators. It is known from the abovementioned results in this research that the correlations among anthropometric indicators are relatively high. Therefore, only the VAI variable was incorporated from the 11 variables for subsequent discussions. Supplementary Table 1 exhibits the baseline characteristics of the training group and validation group, with no statistically salient disparity existing between the two groups (*p* > 0.05). LASSO regression analysis was conducted in the training group. The LASSO regression encompassed 37 clinical variables, and the coefficients and the related log(*λ*) values are displayed in Figure 5. When the λ value was 0.01415317, the minimum cross-validation error was identified, obtaining 15 clinical variables with non-zero coefficients. When the λ value was 0.06882121, the minimum cross-validation error within one standard error was identified, obtaining three clinical variables with non-zero coefficients (Ca^2+^, CRP, FIB).

### 3.6 Assess the predictive efficacy of the model

**Figure 6:**
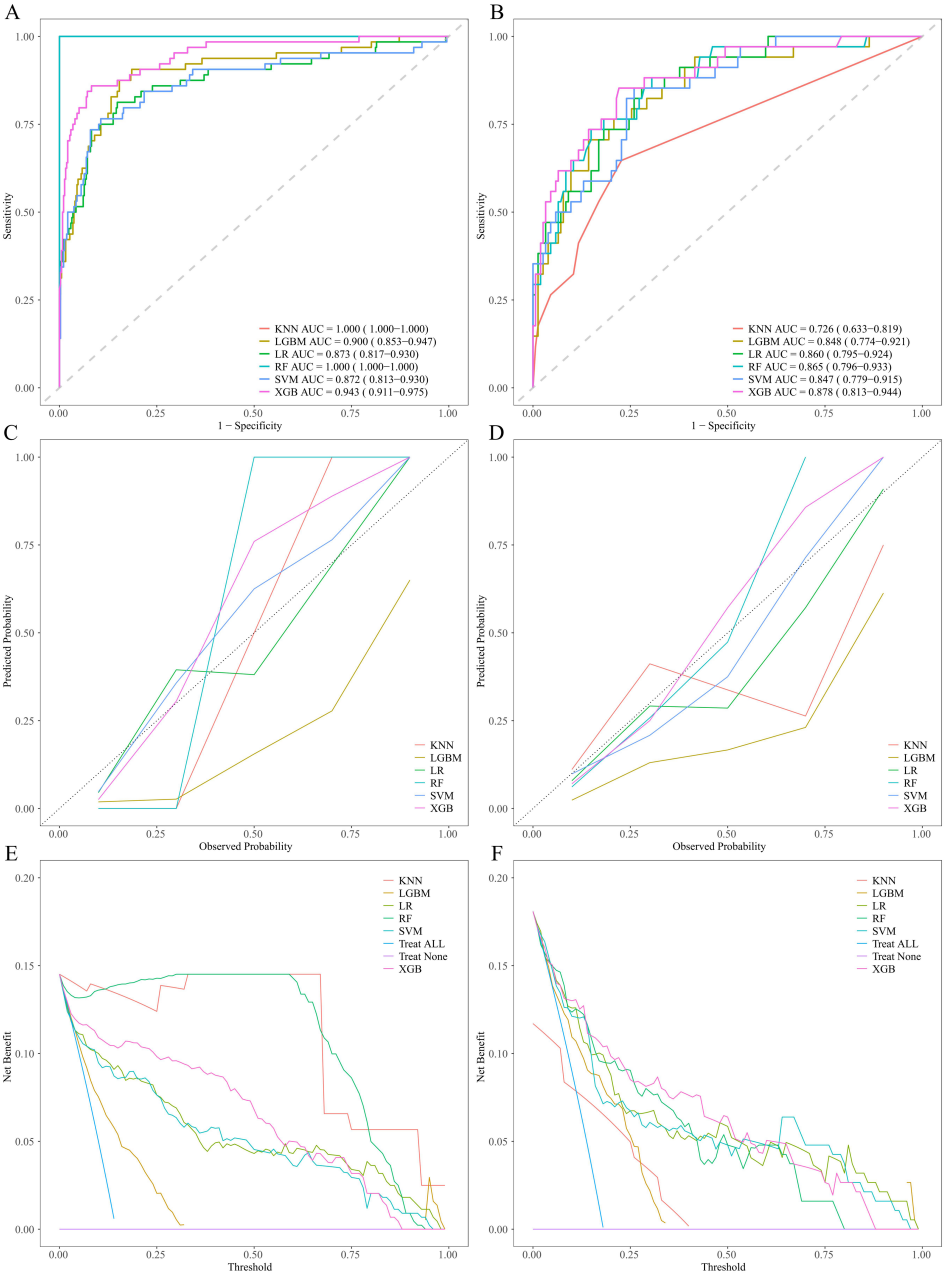
Performance comparison of ML models. ROC curves on the training group **(A)** and validation group **(B)**. Calibration plots on the training group **(C)** and validation group **(D)**. Decision curves on the training group **(E)** and validation group **(F)**. ROC, receiver operating characteristic.

**Figure 7:**
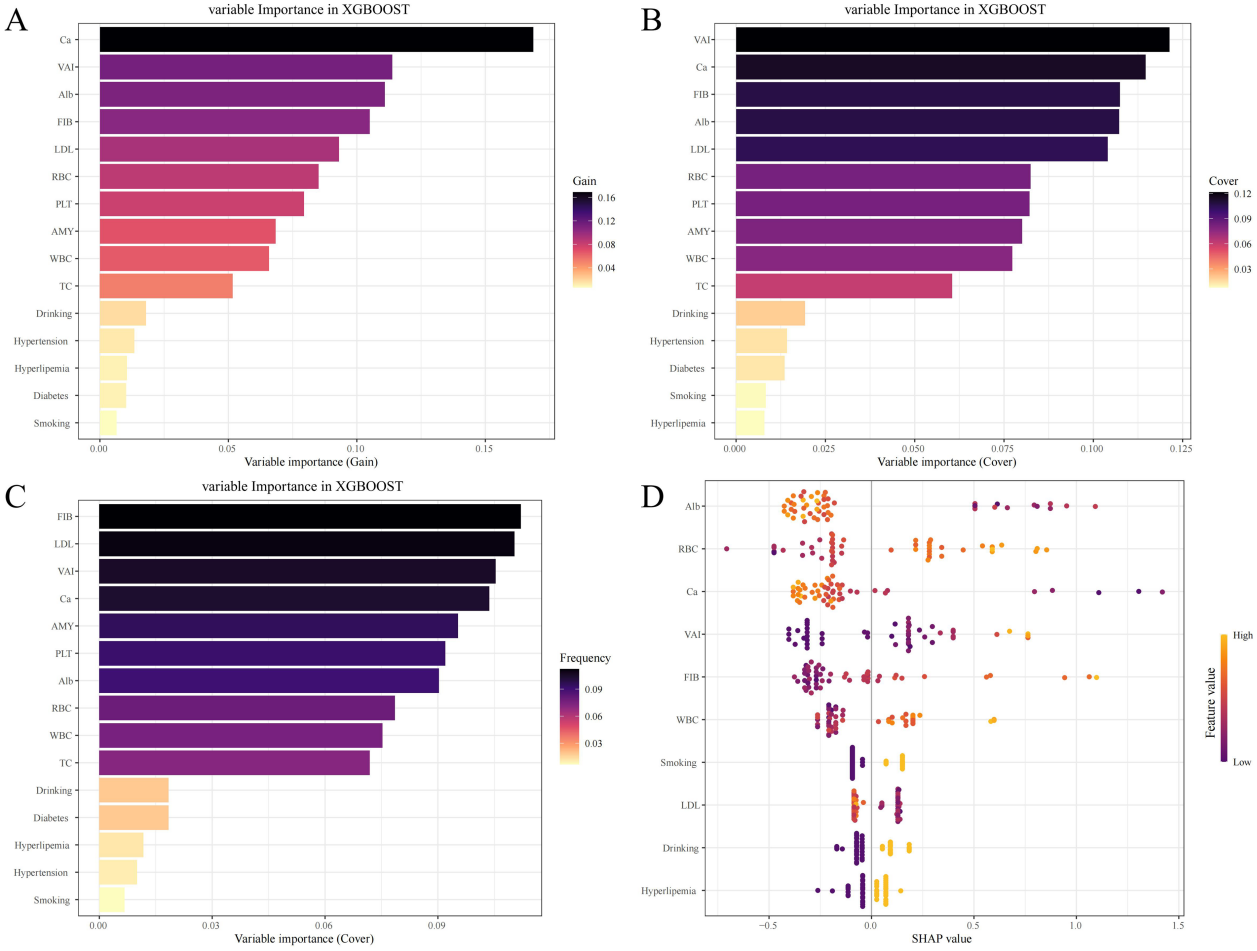
Variable Importance Plots in the XGB Model. **(A)** The Gain Metric; **(B)** The Cover Metri; **(C)** The Frequency Metric; **(D)** Shapley Additive exPlanations visualization.

The aforementioned 15 selected variables were input into ML models (KNN, LGBM, LR, RF, SVM, XGB). The model performances are presented in Table 5 and Figure 6. The key to evaluating the performance of the model lies in its performance on the validation group. In the validation group, the XGB model exhibited the highest AUC. The other models likewise demonstrated favorable AUC, signifying that all the ML models possess excellent discriminatory ability (Table 5). Among them, the XGB model had the highest AUC, accuracy, precision, and F1 score; the LGBM model presented the best sensitivity and negative predictive value; and the RF model presented the highest specificity and positive predictive value. The calibration curves reveal that the XGB model, the SVM model, and the RF model displayed relatively good consistency between the observed and predicted outcomes (Figure 6D). The DCA curves indicate that the XGB model achieved the best DCA results, while the LGBM model manifested inferior DCA results (Figure 6F). To sum up, the XGB model was ultimately selected as the ideal model because it had the highest AUC and a relatively high recall rate in the validation group, along with the largest net benefit and a wide range of high-risk thresholds. The importance graph of the clinical variables constituting the XGB model is presented in Figure 7.

**Table 5:** Performance of machine learning algorithms.

Class	Model	AUC (95% CI)	Accuracy	Sensitivity	Specificity	Pos pred value	Neg pred value	F1 score
Training group								
	KNN	1.000 (1.000–1.000)	1.000	1.000	1.000	1.000	1.000	1.000
	LGBM	0.900 (0.853–0.947)	0.861	0.875	0.846	0.491	0.976	0.629
	LR	0.873 (0.817–0.930)	0.832	0.812	0.851	0.481	0.964	0.605
	RF	1.000 (1.000–1.000)	1.000	1.000	1.000	1.000	1.000	1.000
	SVM	0.872 (0.813–0.930)	0.830	0.766	0.894	0.551	0.957	0.641
	XGB	0.943 (0.911–0.975)	0.889	0.859	0.918	0.640	0.975	0.733
Validation group								
	KNN	0.726 (0.633–0.819)	0.610	0.324	0.896	0.407	0.857	0.361
	LGBM	0.848 (0.774–0.921)	0.764	0.794	0.734	0.397	0.942	0.529
	LR	0.860 (0.795–0.924)	0.757	0.735	0.779	0.424	0.930	0.538
	RF	0.865 (0.796–0.933)	0.677	0.412	0.942	0.609	0.879	0.491
	SVM	0.847 (0.779–0.915)	0.706	0.588	0.825	0.426	0.901	0.494
	XGB	0.878 (0.813–0.944)	0.782	0.706	0.857	0.522	0.930	0.600

AUC, area under the curve; CI, confidence interval; KNN, K-Nearest Neighbor; LGBM, Light Gradient Boosting Machine; LR, Logistic Regression; RF, Random Forest; SVM, Support Vector Machine; XGB, eXtreme Gradient Boosting.

## 4 Discussion

This study shows the correlation between anthropometric indicators of visceral obesity and the severity of AP, and demonstrates that the VAI is the most accurate anthropometric predictor of AP. Additionally, this study constructs an ML model using VAI and other common clinical variables and discovers that the XGB model may be a promising tool for the early prediction of the severity of AP patients.

In AP, mild cases only present with pancreatic edema and have a favorable prognosis (4). Conversely, severe cases may result in multiple OF throughout the body, with a mortality rate as high as 20–40% (5, 6). Therefore, assessing the severity of AP during the early stages of hospitalization is of considerable importance (7). Timely intervention for high-risk patients is a necessary condition for reducing mortality and improving prognosis. The current commonly used clinical scoring systems, on the one hand, are unable to complete the assessment as early as possible, and on the other hand, have complex parameters, thus being limited to a certain extent in clinical application. Furthermore, none of these clinical scoring systems take into account the differences in obesity or the distribution of visceral fat.

In recent years, apart from the traditional Body Mass Index (BMI), several anthropometric indices have been employed to assess fat distribution, such as BRI, CI, LAP, WTI, CMI, VAI, and CVAI. In a cross-sectional study involving tens of thousands of American adults, the BRI index was demonstrated to be a predictive marker for diabetes and prediabetes (35). They have, respectively, been verified and shown certain predictive efficacy in multiple diseases. In a study encompassing three databases, it was discovered that, compared with BMI, BRI is a clinical indicator that is more effective in assessing body fat conditions (21). CI was established as an indicator several decades ago (23). This index was formulated as an indicator for obesity and the distribution of body adiposity. LAP is a crucial indicator for various metabolic disorders, particularly obesity. It was found in a cohort of 1,912 obese adult subjects that LAP and CMI possess superior diagnostic accuracy for metabolic syndrome and can be utilized for the identification of metabolic syndrome (36).

In a study involving 279 women, it was discovered that LAP and VAI can effectively assess visceral fat distribution, indirectly express visceral obesity function. Moreover, they constitute more sensitive indicators for evaluating total body adiposity and fat accumulation in the central abdominal region in contrast to BMI (28). Liu et al. developed the indicator of WTI and discovered through cross-sectional studies that WTI presents differences in detecting metabolic syndrome in women and is a suitable marker for screening metabolic syndrome in population studies (26). A nationwide Chinese study of 60,000 participants found that WTI strongly predicted hyperuricemia (37). A study involving 47,683 adults in China found that CMI serves as a dependable new marker for identifying the metabolic obesity phenotype among individuals with normal weight (27). Research has shown that CVAI serves as a dependable and useful biomarker for assessing visceral adiposity dysfunction in the Chinese population, and it can also be employed to evaluate the metabolic health status of individuals in Asia (29).

A Chinese cohort of 3 million participants identified CVAI as a robust predictor of all-cause, cardiovascular, and cancer mortality (38). This finding underscores its potential value in health economic planning and secondary prevention. Currently, there is a lack of studies examining the relationship between anthropometric measurements and the severity of AP. So far, only prior research has addressed the link between these body metrics and the severity of HLAP (31). However, relevant studies on AP of all etiologies are lacking.

This study highlights the relationship between anthropometric indicators and AP in various disease patterns. This study explored the relationships between 11 anthropometric indicators (WC, BRI, BMI, ABSI, CI, WWI, LAP, WTI, CMI, VAI, and CVAI) and the severity of AP, and discovered that VAI, CMI, LAP, and WTI were independent predictors of the severity of AP. There existed a significant correlation among these four indicators, and they exhibited higher diagnostic capabilities compared to other anthropometric methods and common clinical scoring systems, providing a direction for risk prediction of the severity of AP. Zhu et al. verified the significant correlations between the indicators of VAI, CMI, and LAP and the severity of HLAP (31). These indicators, especially VAI, demonstrated the highest predictive capacity and were conducive to the prediction and assessment of the severity of HLAP. This is analogous to the results of this study. Nevertheless, although ABSI and WWI are anthropometric indicators of visceral obesity, they did not demonstrate a significant correlation with the severity of AP. To further validate the predictive capacity of the indicators, in this study, VAI and other common clinical indicators were employed to construct six ML models. As the indicators incorporated into the models were all routine examinations for AP patients, the constructed models were convenient and rapid, facilitating their application by clinicians in clinical practice. Furthermore, the models in this study exhibited relatively superior predictive accuracy. The results revealed that the XGB model presented the highest AUC in the validation group, along with relatively favorable calibration curves and DCA results. Consistent with our validation results showing AUC = 0.878, the XGB model is a promising tool for the early assessment of the severity of AP patients and possesses good clinical applicability.

This research showcases multiple strengths. Most importantly, it represents the first study to explore the relationship between AP and anthropometric indicators in a group sourced from a major tertiary hospital within the Chinese population. Secondly, the anthropometric indicators examined in this study can act as a cost-effective alternative approach for the early assessment of the severity of AP during hospitalization in specific patients (for instance, patients who are unable to obtain clinical scoring systems due to physical factors), which is more expeditious and convenient than traditional clinical scoring and can facilitate the application of clinicians in clinical practice. It showcases superior predictive accuracy. Among them, the predictive efficacy of VAI, CMI, LAP, and WTI is more outstanding than that of Ranson score, Glasgow score, BISAP, and APACHE II score, while showing a comparable predictive capacity to that of the SIRS score. Nonetheless, this study is not devoid of limitations. It is a retrospective analysis conducted at one institution, which may be susceptible to both selection bias and detection bias. Therefore, it should be interpreted cautiously. More extensive multicenter studies involving larger participant cohorts are warranted to validate the true predictive value of anthropometric indicators.

## 5 Conclusion

VAI, CMI, LAP, and WTI are independent predictors of AP severity, with VAI showing the highest individual predictive capability among them. The XGB model, incorporating VAI and routinely available clinical variables, achieved excellent performance (AUC = 0.878) for early severity assessment, offering a potentially rapid and cost-effective clinical tool. This supports the utility of visceral obesity anthropometric indicators and ML models for improving early risk stratification in AP.

## Data Availability

The data analyzed in this study is subject to the following licenses/restrictions: the datasets used and analyzed during the current study are available from the corresponding author on reasonable request. Requests to access these datasets should be directed to Dingzhou Wang, wdz199802@163.com.

## Glossary

AP: Acute pancreatitis
MAP: Mild acute pancreatitis
MSAP: Moderately severe acute pancreatitis
SAP: Severe acute pancreatitis
SIRS: Systemic inflammatory response syndrome
BISAP: Bedside index of severity in acute pancreatitis
APACHE II: Acute physiology and chronic health evaluation II
JSS: Japanese severity score
BMI: Body mass index
BRI: Body roundness index
ABSI: A body shape index
WWI: Weight-adjusted waist index
LAP: Lipid accumulation products
WTI: Waist triglyceride index
CMI: Cardiometabolic index
VAI: Visceral adiposity index
CVAI: Chinese visceral adiposity index
OF: Organ failure
WC: Waist circumference
WBC: White blood cell
RBC: Red blood cell
Hb: Hemoglobin
PLT: Platelet counts
HCT: Hematocrit value
TBil: Total bilirubin
Alb: Albumin
ALT: Alanine aminotransferase
AST: Aspartate transaminase
ALP: Alkaline phosphatase
BG: Blood glucose
BUN: Blood urea nitrogen
Cr: Creatinine
K^+^: Potassium
Na^+^: Sodium
Cl^−^: Chlorine
Ca^2+^: Calcium
AMY: Amylase
TC: Total cholesterol
TG: Triglyceride
HDL: High-density lipoprotein cholesterol
LDL: Low-density lipoprotein cholesterol
LIP: Lipase
CRP: C-reactive protein
TT: Thrombin time
PT: Prothrombin time
Fib: Fibrinogen
APTT: Activated partial thromboplastin time
INR: International standardized ratio
ML: Machine learning
LASSO: Least Absolute Shrinkage and Selection Operator
KNN: K-Nearest Neighbor
LGBM: Light Gradient Boosting Machine
LR: Logistic Regression
RF: Random Forest
SVM: Support Vector Machine
XGB: eXtreme Gradient Boosting
ROC: receiver operating characteristic
AUC: area under the ROC curve
DCA: decision curve analysis
