# Supplementary material for: Visceral obesity anthropometric indicators as predictors of acute pancreatitis severity
Source: Front Med (Lausanne). 2025 Jul 11;12:1536090. doi: 10.3389/fmed.2025.1536090 (PMC12289579; doi:10.3389/fmed.2025.1536090)
Supplement: Supplementary file 1 [file Table_1.docx]

Supplementary Material

**Supplementary Table 1.** Characteristics of the training and validation groups

| **Variables** | **Training group (n=441)** | **Validation group (n=188)** | ***p* value** |
| --- | --- | --- | --- |
| **Baseline characteristics** |  |  |  |
| Gender, male, N (%) | 309 (70.07) | 128 (68.09) | 0.689 |
| Age, years (IQR) | 47.00 (38.00, 58.00) | 46.00 (37.00, 59.00) | 0.884 |
| BMI, kg/m^2^ (IQR) | 25.18 (22.96, 27.68) | 24.87 (22.42, 27.72) | 0.358 |
| WC, cm (IQR) | 85.38 (78.95, 91.22) | 85.13 (78.40, 91.62) | 0.982 |
| VAI (IQR) | 2.73 (1.30, 7.76) | 2.65 (1.17, 11.28) | 0.612 |
| MSAP/SAP, N (%) | 64 (14.51) | 34 (18.09) | 0.312 |
| **Medical history** |  |  |  |
| Hypertension, N (%) | 91 (20.63) | 48 (25.53) | 0.211 |
| Diabetes, N (%) | 113 (25.62) | 54 (28.72) | 0.479 |
| Fatty Liver, N (%) | 265 (60.09) | 107 (56.91) | 0.514 |
| Hyperlipemia, N (%) | 191 (43.31) | 91 (48.40) | 0.276 |
| Drinking, N (%) | 179 (40.59) | 74 (39.36) | 0.843 |
| Smoking, N (%) | 151 (34.24) | 73 (38.83) | 0.313 |
| **Etiology** |  |  | 0.832 |
| Biliary, N (%) | 184 (41.72) | 79 (42.02) |  |
| Hyperlipemia, N (%) | 31 (7.03) | 10 (5.32) |  |
| Alcohol abuse, N (%) | 141 (31.97) | 59 (31.38) |  |
| Unknown, N (%) | 85 (19.27) | 40 (21.28) |  |
| **Laboratory findings** |  |  |  |
| WBC, 10^9^/L (IQR) | 10.23 (7.68, 13.34) | 10.34 (7.64, 13.73) | 0.888 |
| RBC, 10^12^/L (IQR) | 4.55 (4.16, 4.97) | 4.59 (4.20, 4.94) | 0.813 |
| Hb, g/L (IQR) | 140 (128, 152) | 139 (126.75, 152) | 0.616 |
| PLT, 10^9^/L (IQR) | 203 (170, 243) | 205 (172, 240) | 0.960 |
| HCT (IQR) | 0.41 (0.38, 0.44) | 0.41 (0.37, 0.45) | 0.659 |
| TBil, μmol/L (IQR) | 20 (15, 29) | 20 (14, 31) | 0.960 |
| Alb, g/L (SD) | 37.66 ± 4.32 | 37.27 ± 4.85 | 0.342 |
| ALT, U/L (IQR) | 31 (18, 77) | 28.50 (17, 76) | 0.447 |
| AST, U/L (IQR) | 28 (19, 60) | 29 (20, 56.25) | 0.695 |
| ALP, U/L (IQR) | 83 (68, 110) | 82 (67, 103) | 0.646 |
| BG, mmol/L (IQR) | 7.4 (5.8, 10.6) | 8.2 (5.9, 11.8) | 0.090 |
| BUN, mmol/L (IQR) | 4.0 (3.0, 5.2) | 3.9 (3.1, 5.3) | 0.586 |
| Cr, μmol/L (IQR) | 65 (54, 76) | 64 (53, 77) | 0.795 |
| K^+^, mmol/L (IQR) | 3.92 (3.69, 4.11) | 3.94 (3.75, 4.15) | 0.308 |
| Na^+^, mmol/L (IQR) | 137.00 (135.00, 139.00) | 137.00 (135.00, 139.00) | 0.673 |
| Cl^-^, mmol/L (IQR) | 103 (100, 105) | 102 (100, 105) | 0.26 |
| Ca^2+^, mmol/L (IQR) | 2.17 (2.07, 2.24) | 2.15 (2.01, 2.25) | 0.335 |
| AMY, U/L (IQR) | 151 (74, 343) | 168 (79, 495) | 0.107 |
| TC, mmol/L (IQR) | 5.36 (4.31, 7.14) | 5.18 (4.17, 7.05) | 0.716 |
| TG, mmol/L (IQR) | 1.95 (0.96, 5.50) | 1.88 (0.92, 6.41) | 0.836 |
| HDL, mmol/L (IQR) | 1.09 (0.88, 1.31) | 1.06 (0.80, 1.32) | 0.505 |
| LDL, mmol/L (IQR) | 3.08 (2.37, 3.89) | 2.96 (2.31, 3.91) | 0.517 |
| LIP, IU/L (IQR) | 193 (71, 445) | 187 (88, 602) | 0.150 |
| CRP,mg/L (IQR) | 118.0 (34.2, 187.6) | 103.8 (45.9, 187.8) | 0.853 |
| TT, seconds (IQR) | 16.3 (15.5, 17.1) | 16.4 (15.7, 17.1) | 0.309 |
| PT, seconds (IQR) | 13.8 (13.2, 14.4) | 13.9 (13.2, 14.5) | 0.635 |
| Fib, g/L (IQR) | 4.85 (3.79, 6.36) | 4.81 (3.68, 6.45) | 0.826 |
| APTT, seconds (IQR) | 37.0 (34.3, 40.3) | 37.4 (34.0, 40.3) | 0.977 |
| INR (IQR) | 1.06 (1.00, 1.12) | 1.06 (1.01, 1.13) | 0.565 |
| **Endpoint event** |  |  |  |
| AICU, N (%) | 10 (2.27) | 6 (3.19) | 0.581 |
| Hospital LOS, N (%) | 6.81 (5.09, 9.64) | 7.70 (5.65, 10.60) | 0.122 |

VAI: Visceral adiposity index; AP: Acute pancreatitis; MAP: Mild acute pancreatitis; MSAP: moderately severe acute pancreatitis; SAP: Severe acute pancreatitis; BMI: Body mass index; WC: Waist circumference; WBC: White blood cell; RBC: Red blood cell; Hb: Hemoglobin; PLT: Platelet counts; HCT: Hematocrit value; TBil: Total bilirubin; Alb: Albumin; ALT: Alanine aminotransferase; AST: Aspartate transaminase; ALP: Alkaline phosphatase; BG: Blood glucose; BUN: Blood urea nitrogen; Cr: Creatinine; K^+^: Potassium; Na^+^: Sodium; Cl^-^: Chlorine; Ca^2+^: Calcium; AMY: Amylase; TC: Total cholesterol; TG: Triglyceride; HDL: High-density lipoprotein cholesterol; LDL: Low-density lipoprotein cholesterol; LIP: Lipase; CRP: C-reactive protein; TT: Thrombin time; PT: Prothrombin time; Fib: Fibrinogen; APTT: Activated partial thromboplastin time; INR: International standardized ratio; AICU: Admission to intensive care unit; LOS: Length of stay.
